# Polygenic Risk Score Analysis of 37 SNPs Associated with Melanoma Risk in Colombian Population

**DOI:** 10.3390/ijms26104674

**Published:** 2025-05-14

**Authors:** David Tovar-Parra, Luz Dary Gutiérrez-Castañeda

**Affiliations:** 1 General Dermatology Group, Hospital Universitario Centro Dermatologico Federico Lleras Acosta E.S.E, Bogotá 111511, Colombia; david.tovar@inrs.ca; 2 Institut National de la Recherche Scientifique INRS, Centre Armand-Frappier Santé Biotechnologie, Laval, QC H7V 1B7, Canada; 3 Research Institute, Basic Health Sciences Group, Fundación Universitaria de Ciencias de la Salud (FUCS), Bogotá 111221, Colombia

**Keywords:** melanoma, polygenic risk score, polymorphism

## Abstract

Melanoma incidence is increasing, with distinct genetic and clinical patterns observed in the Latin American population. This study aimed to evaluate melanoma risk in a Colombian cohort through polygenic risk analysis using 37 variants across nine genes previously associated with melanoma. We performed polygenic risk score (PRS) analysis on 85 melanoma patients and 165 controls. Genotyping was performed for 37 melanoma-associated SNPs, and on the basis of previous GWAS reports, individual PRSs were calculated for each participant. The participants were then stratified into quartiles to examine risk gradients. In addition, phenotypic features such as eye and hair color were evaluated, and genetic models and haplotype analyses were performed, adjusting for sex and family history of cancer. PRS quartile stratification revealed a clear risk gradient. Notably, 31.8% of the melanoma cases were clustered in the highest-risk quartile (Q4), with a maximum PRS of 1.04. Variants in *TYR*, *TYRP1*, *CDKN2A*, and *HERC2* significantly contributed to risk, and light brown eye and hair colors were strongly associated with increased melanoma risk. Moreover, a protective haplotype in the *OCA2-HERC2* region was identified among males. The integration of the PRS with clinical and phenotypic factors has potential for improving melanoma risk stratification in the Colombian population, warranting further investigation in larger, diverse cohorts.

## 1. Introduction

Several factors contribute to melanoma development, including environmental factors (such as exposure to ultraviolet radiation), clinical characteristics (age, sex, phototype, nevus count, freckling, hair, and skin color), and genetic predispositions (mutations, copy number variations, and germline variants) [1,2,3]. Notably, the incidence of melanoma has been increasing in Latin America over the past few decades [4]. In Colombia, incidence rates range from 2 to 4 per 100,000 habitants, with a higher prevalence in females than in males [5,6]. However, according to the National Cancer Institute of Colombia, incidence rates vary between 0.4 and 9.3 per 100,000 in females and between 0.1 and 6.3 per 100,000 in males [7,8].

Genetic factors play crucial roles in melanoma development, contributing to up to 50% of cases [9]. Melanoma arises from somatic mutations that trigger the malignant transformation of melanocytes, including mutations in *BRAF*, *NRAS*, *TERT*, *PI3K*, and *KIT*, among other genes [10,11]. However, germline variants have also been associated with susceptibility to melanoma [12,13]. Germline variants in *CDKN2A*, *CDK4*, *BAP1*, *POT1*, *ACD*, *TERF2IP*, and *TERT* have been identified as high-penetrance variants associated with melanoma development [14]. Additionally, variants in *MC1R*, *MITF*, and *SLC45A2* are considered moderate-penetrance variants, whereas germline variants in *TYR*, *OCA2*, *ASIP*, *PLA2G6*, *FTO*, *PARP1*, *ATM*, *CDKAL1*, *CCND1*, and *CYP1B1* are classified as low-penetrance variants [15,16]. Although these low-penetrance variants alone do not necessarily lead to melanoma development, they contribute to polygenic susceptibility, and their interactions may significantly increase melanoma risk [17].

Over the years, approximately 1140 variants in nearly 260 genes have been associated with early-onset melanoma across different populations [18,19]. The combined analysis of these variants has emerged as a valuable tool for estimating risk beyond traditional phenotypic and clinical characteristics [20]. The polygenic risk score (PRS) integrates the cumulative effects of multiple genetic variants across the genome to generate a personalized risk estimate for disease development, making it a useful tool for melanoma screening and early detection [21].

Recently, studies have highlighted the importance of considering genetic ancestry when selecting SNPs for PRS construction. In 2016, Ossa et al. described the ancestry landscape of the Colombian population, reporting 57% European and a substantial contribution from Native American ancestries in the Andes region [22]. In contrast, Rishishwar et al. found an ancestry composition of 74% European, 18% Native American, and 7% African [23]. This admixture may influence the frequency of melanoma subtypes: while superficial spreading melanoma (SSM) predominates in European and American populations (60–70%) [24,25], Latin American populations show a higher frequency of acral lentiginous melanoma (ALM), with reports of 44.1% in Mexico [26], 61.2% in Peru [27], and 43.7% in Colombia, among other variations [28,29,30,31].

Variants in *CDKN2A*, *CDK4*, and *MC1R*—such as G101W, A148T, and R24C—have been associated with melanoma in populations from Poland [32], Spain [33], and the United Kingdom (UK) [34] as well as in Brazilian [35,36] and Austrian populations [37]. In our previous study, haplotype analyses of *CDKN2A* and *SLC45A2* were significantly associated with melanoma risk in Colombian patients [12,38,39]. Given the complex genetic landscape, evaluating a single variant is insufficient to capture melanoma risk in a representative Colombian cohort. Therefore, the use of a PRS that combines multiple genetic variants is warranted.

Recent applications of PRS in melanoma risk assessment further support this approach. For instance, Potjer et al. determined a PRS using 46 SNPs in a Dutch population, finding a significant association with melanoma risk [9]. In 2024, Pellegrini et al. demonstrated that a PRS based on 57 SNPs improved melanoma risk assessment in an Italian cohort [40]. Additionally, emerging PRS models based on SNP selections from various GWAS—primarily conducted in European and Australian populations—are refining risk prediction strategies [21].

A recent GWAS meta-analysis, which included populations from the United Kingdom, United States, Australia, and Northern and Western Europe, examined 36,760 melanoma cases and 375,188 controls, identifying 54 significant loci with 68 independent SNPs [21,41]. Among these, several SNPs were located near or within genes associated with pigmentation (*ASIP*, *SLC45A2*, *HERC2*/*OCA2*, *MC1R*, and *TYR*) or linked to nevus count and familial melanoma (*TERT*, *PLA2G6*, *CDKN2A*-*MTAP*, and *IRF4*) [42]. However, no GWASs have been conducted in Colombian or broader Latin American populations. To address this gap, we evaluated 37 SNPs in genes previously associated with melanoma development in a cohort of the Colombian population [12,38,39].

## 2. Results

Among the 250 participants, 85 were melanoma patients, while the remaining 166 were healthy individuals. The demographic and clinical characteristics of both the case and control groups have been previously reported [12].

### 2.1. Polygenic Risk Score (PRS) Distribution Between Cases and Controls

**Figure 1:**
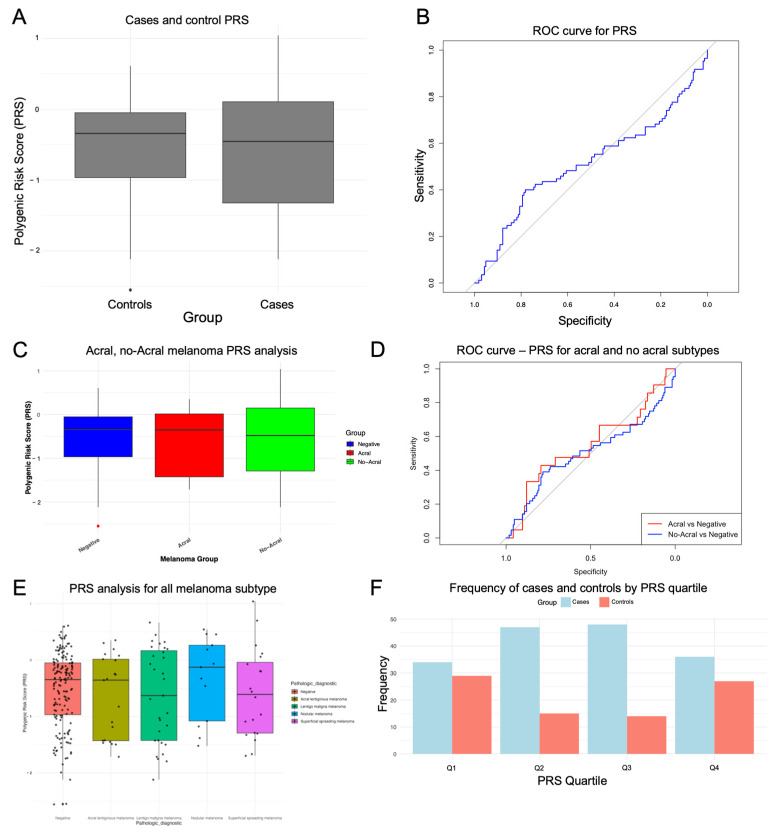
Polygenic risk score (PRS) calculation and distribution by cutaneous melanoma risk. (**A**) PRSs for controls and cases with medians and SDs; (**B**) graphical representation of the area under the curve (AUC) for predicting the risk of melanoma development with the PRS37; (**C**) PRSs for acral and no-acral melanoma with medians and SDs; (**D**) graphical representation of the area under the curve (AUC) for predicting the risk of melanoma development with acral and no-acral melanoma; (**E**) PRSs for all melanoma subtypes with medians and SDs; (**F**) frequency of cases and controls by PRS quartiles.

For each participant, an individual PRS was calculated. Among the melanoma patients, the PRS values ranged from −2.11 to 1.041, with a mean of −0.57 (Supplementary Table S3). In the controls, the PRS ranged from −2.55 to 0.61, with a mean of −0.52 (Supplementary Table S3) (Figure 1A). No significant differences were observed between cases and controls regarding PRS (*p* = 0.568, AUC = 0.5154) (Figure 1B). For the predictive models using PRS alone and combined with age and sex, we did not see an improved AUC-ROC, with a value of 324.15 compared to 327.94, respectively (Supplementary Table S3). The logistic regression model assessing the association between the polygenic risk score (PRS) and melanoma status revealed that PRS was not a significant predictor (β = −0.114, SE = 0.190, z = −0.603, *p* = 0.546). The model’s Nagelkerke pseudo-R^2^ was 0.00145, indicating that the panel of 37 SNPs accounted for approximately 0.15% of the phenotypic variance in melanoma risk (Supplementary Table S3).

A PRS analysis was also conducted for different melanoma subtypes, stratifying cases into acral and non-acral melanomas to investigate possible interactions between SNPs associated with pigmentation. In both models, PRS was not significant, with a PRS of −0.28 (*p* = 0.4) and an AUC = 0.54 for the acral melanoma model, and a PRS of −0.06 (*p* = 0.754) with an AUC = 0.504 for the non-acral melanoma model, indicating no predictive capability (Figure 1C,D, Supplementary Table S3). When analyzing PRS for each melanoma subtype separately, we observed that the estimated PRS for nodular melanoma was 1.64, that for superficial spreading melanoma was 0.86, for that lentigo maligna melanoma was 0.81, and that for acral lentiginous melanoma was 0.75, which exhibited the lowest risk. Although the PRS for nodular melanoma was 1.64, it was not statistically significant (*p* = 0.31) (Figure 1E). Additionally, AUC values ranging from 0.52 to 0.59 suggest that the model does not effectively discriminate between melanoma cases and controls (Supplementary Table S3).

To account for PRS variability, participants were stratified into four quartiles. Quartile 1 (Q1) represents the lowest PRS values, with a mean of −1.52 (SD = 0.291, range = −2.56 to −1.10), whereas Quartile 4 (Q4) represents the highest PRS values, with a mean of 0.264 (SD = 0.197, range = 0.0106 to 1.04). Statistical analysis confirmed significant differences in PRS across quartiles, as one-way ANOVA yielded an F value of 810 (*p* < 2 × 10^−16^) (Supplementary Table S5). These results validated the quartile-based stratification and demonstrated a clear gradient in PRS values (Figure 1F). This quartile-based stratification was used to assess the distribution of PRS within both melanoma patients and controls. In terms of quartile distribution, 34.1% of the melanoma patients and 20.6% of the controls fell into Q1. In Q2, 17.6% of the cases and 28.5% of the controls were observed. Q3 included 16.5% of the cases and 29.1% of the controls, whereas Q4 comprised 31.8% of the cases and 21.8% of the controls (Table 1). Although the quartile-based analysis revealed significant differences in PRS between groups, the primary goal was to visualize and explore the distribution of genetic risk across varying levels of PRS within the sample. This stratification approach provides valuable insight into how genetic risk, as measured by the PRS, is distributed in both melanoma patients and healthy controls, even though the overall PRS failed to significantly differentiate between the two groups in the logistic regression model.

### 2.2. Participant Characteristics According to the Quartile Distribution in Cases and Controls

Participants were classified into quartiles based on individual PRSs (Supplementary Tables S4 and S5). In Q1, 58% of the cases and 52% of the controls were female. In Q2, females accounted for 46% of the cases and 55% of the controls, whereas in Q3, females accounted for 50% of the cases and 60% of the controls. A shift was observed in Q4, where 51% of the cases and 44% of the controls were female.

With respect to phototype distribution, skin phototype III was the most common type across both cases and controls (50–76%). However, in Q3, phototype III accounted for 50% of the cases and 72% of the controls (Table 2). Regression analysis comparing clinical characteristics across quartiles revealed associations with eye and hair color. Light brown eye color was identified as a risk factor in Q4, with an odds ratio (OR = 5.5, CI = 1.51–20.02, *p* = 0.0063), whereas black eye color was a protective factor (OR = 0.30, CI = 0.10–0.91, *p* = 0.0063). Similar findings were observed for hair color, where black hair was protective (OR = 0.01, CI = 0.01–0.36, *p* = 0.0002), whereas light brown hair was associated with increased risk (OR = 11.68, CI = 2.31–59.03, *p* = 0.001) (Table 3).

In terms of pathological characteristics by quartile, acral lentiginous melanoma and lentigo maligna melanoma were the most frequent subtypes in Q1 (~30%). In Q2, superficial spreading melanoma predominated (~40%), whereas lentigo maligna melanoma was most common in Q4. The Clark level of >4 mm increased from 33% in Q2 to 55% in Q4. However, no differential distribution was observed for Breslow thickness across quartiles. In terms of melanoma localization, 52% of melanomas in Q4 were located on the head and neck, followed by 22% on the hands and feet (Table 2).

### 2.3. Distribution of Genetic Variants Across PRS Quartiles

Quartiles were defined based on individual PRS values, considering the cumulative effect of 37 SNPs per patient. For Q4 (highest PRS), the SNPs with the greatest impact on the individual score included rs3088440 (*CDKN2A*-540—3′UTR), rs1126809 (*TYR*—p.R402Q), rs1042602 (*TYR*—p.S192Y), rs916977 (*HERC2*—Intron 12), rs7170852 (*HERC2*—Intron 56), rs12913832 (*HERC2*—Intron 86), and rs885479 (*MC1R*—p.R163Q) (Supplementary Table S6).

### 2.4. Associations Between Genetic Variations and Melanoma Risk

Among the 37 SNPs analyzed across the *CDKN2A*, *CDK4*, *SCL45A2*, *MITF*, *TYR*, *TYRP1*, *OCA2*, *HERC2*, and *MC1R* genes, 11 variants in *CDKN2A*, *CDK4*, and *SCL45A2* have been previously reported [12,38,39]. The remaining 26 variants were analyzed via multiple genetic models, including dominant, codominant, recessive, log-additive, and overdominant models, which were applied only when the risk allele was present in the wild-type, heterozygous, and homozygous states. For the *TYR* variants (rs1042602–p.S192Y and rs1126809–p.R402Q), rs1042602 was associated with melanoma risk in both the codominant and recessive models (OR = 3.32, CI = 1.01–10.94, *p* = 0.032) (Table 4).

With respect to *TYRP1* variants (rs281865424–p.R356E and rs1408799), rs1408799 was identified as a melanoma risk factor under the log-additive model (OR = 2.29, CI = 1.07–4.90, *p* = 0.027). Among the *HERC2* variants (rs916977–intron 12, rs7170852–intron 56, and rs12913832–intron 86), only rs7170852 was associated with melanoma risk according to the homozygous dominant and recessive models (OR = 12.22, CI = 1.48–100.91, *p* < 0.0001; OR = 21.92, CI = 2.73–176.14, *p* < 0.0001, respectively). Conversely, in the heterozygous codominant, dominant, and overdominant models, rs7170852 was protective (OR = 0.28, 0.52, and 0.20; *p* = 0.04 and <0.0001, respectively) (Table 4).

Conversely, no associations were detected between variants rs7497270 and rs1800407 (p.R419Q) of the *OCA2* gene, or between variants rs1805005 (p.V60L), rs885479 (p.R163Q), and rs1805009 (p.D294H) of the *MC1R* gene, with either a protective or risk effect in any of the genetic models analyzed (Supplementary Table S7).

### 2.5. OCA2-HERC2 and MC1R Haplotype Frequencies

For the haplotype analysis, two independent analyses were performed. First, we conducted a haplotype analysis adjusted for sex and family history of cancer for the variants of the *OCA2* and *HERC2* genes and a separate analysis for the variants of the *MC1R* gene. In the haplotype analysis of *OCA2* (rs7497270, rs1800401, rs1800407, and rs1800414) and *HERC*2 (rs916977, rs7170852, and rs12913832), we observed that the TCCTTTA haplotype in males was associated with a protective effect against melanoma (OR = 0.14, 95% CI = 0.02–0.96). Conversely, in the haplotype analysis of the *MC1R* gene, no significant association was found between haplotypes and melanoma, either as a risk or protective factor (Supplementary Table S8). The linkage disequilibrium (LD) analysis revealed varying degrees of genetic association among the studied SNPs in *OCA2* and *HERC2*. The D’ statistic indicated a strong linkage between *OCA2*_Exon9 and *OCA2*_Exon12 (D’ = 0.8995), as well as between *HERC2*_rs916977 and *HERC2*_rs12913832 (D’ = 0.8736). However, the r statistic showed weaker correlations, suggesting partial recombination between these loci. Additionally, the LD analysis of *MC1R* variants demonstrated high D’ values for several SNP pairs, including V60L-D84E (D’ = 0.9197) and V60L-V92M (D’ = 0.982), indicating strong linkage. Despite this, the r values were lower, reflecting limited correlation in allele frequencies (Supplementary Table S8).

## 3. Discussion

We performed polygenic risk scoring on 250 participants via the polymorphic characteristics of 37 SNPs that have been previously associated with melanoma susceptibility in various populations. When analyzed as a continuous variable, the overall PRS distribution did not differ significantly between melanoma patients and controls. However, quartile-based stratification revealed a clear risk gradient. Notably, 31.8% of the melanoma cases fell into the highest risk quartile (Q4), where the maximum PRS reached 1.04. Additionally, phenotypic traits such as light brown eye and hair color were strongly associated with increased melanoma risk, with odds ratios (ORs) of 5.50 and 11.68, respectively.

Given that our participants were previously screened for several pathogenic variants in known high-risk, high-penetrance susceptibility genes, these findings provide insight into the aggregate effects of multiple genetic variants in the population [12,38,39]. Specific variants in the *TYR*, *TYRP1*, *CDKN2A*, and *HERC2* genes emerged as key contributors to the elevated risk observed in Q4, whereas haplotype analysis revealed a protective effect of the TCCTTA haplotype in the *OCA2-HERC2* region among males.

The risk allele frequencies, *p* values, and effect sizes for these SNPs were derived from previous GWASs conducted in European, North American, Australian, and United Kingdom populations, where numerous risk loci have been identified [43,44,45]. For example, a GWAS meta-analysis by Landi et al., which included 36,760 melanoma cases and 375,188 controls, identified 85 significant melanoma susceptibility loci and 68 independent SNPs across several genes, including *TERT*, *AGR3*, *CDKN2A*, *OCA2*, *MC1R*, *TP53*, *SLC45A2*, *IRF4*, *CCND1*, *GPRC5A*, and *FTO* [41]. However, these variants did not show significance in the Colombian population. It is possible that genetic admixture and geographic factors related to the equatorial region influence melanoma susceptibility. The Colombian population is characterized by a unique genetic admixture comprising 34–36% Indigenous Americans, 7–10% Africans, and 53–58% individuals European ancestry, which may result in different allele frequencies and risk associations [22,23,46].

The quartile stratification performed in this study effectively highlighted subtle genetic risk gradients attributable to SNPs such as rs3088440, rs1126809, rs1042602, rs916977, rs7170852, rs12913832, and rs885479. Previous studies, such as those by Maccioni et al. and Barrett et al., reported an association between rs3088440 and melanoma risk in the analysis of 837 cases and 1154 controls [47], as did rs3088440 and rs1126809 in 5.374 cases and 7.691 controls from the European population [48]. Similarly, Zhang et al. used a GWAS in almost 10,183 participants and reported that rs12913832 was associated with both brown eye color and melanoma risk in European Americans [49]. This quartile stratification analysis of aggregate SNPs identified some of the most influential variants and revealed subtle genetic risk gradients that might be overlooked in conventional analyses.

The integration of PRSs into melanoma risk prediction models offers a promising avenue for early detection, particularly in understudied Colombian populations. Our results suggest that combining an individual’s PRS with traditional clinical risk factors such as skin phototype, sex, and eye and hair color could increase the accuracy of risk stratification. Although the PRS approach in combination with clinical factors offers improved risk prediction, its implementation as a screening tool in populations with low melanoma incidence (2–4 per 100,000 inhabitants) requires further cost-effectiveness analysis and validation in larger cohorts.

In our study, light brown eye and hair colors were linked to a greater risk of melanoma (OR = 5.50, 95% CI = 1.51–20.02 and OR = 11.68, 95% CI = 2.31–59.03, respectively), whereas black or dark brown features appeared protective (OR= 0.30 and 0.07, respectively). These observations are in line with previous reports, including the findings of Landi et al., who noted differences in the distribution of the hair color PRSs for acral lentiginous melanoma compared with the nonacral subtype [41]. In addition, Gu et al. reported that PRS values—along with eye color, hair color, and skin phototype—were associated with an increased risk of melanoma in Greek, Italian, and Spanish populations [42]. Their analysis incorporated various PRS modeling approaches, including PRS + demographic and PRS + demographics + pigmentation and nevi [42]. However, studies conducted by Wong et al. have not established a correlation between PRS and clinical characteristics in a cohort of 500,000 participants from the United Kingdom [20]. The identification of individuals in high-risk quartiles could facilitate targeted screening and preventive interventions. However, our data and previous reports support the notion that polygenic models are more effective in capturing the multifactorial nature of melanoma risk than single-gene analyses are.

The variants identified in our study have known roles in pigmentation, melanocyte biology, and nevus formation [41,45,50]. For example, TYR variants (rs1042602 and rs1126809) have been implicated in melanin synthesis [51]. We found that the rs1042602 SNP was associated with a risk factor in both the dominant and recessive genetic models, with ORs of 3.44 for the dominant model and 3.32 for the recessive model (*p* = 0.032). Additionally, data reported by Ibarrola-Villava et al., who analyzed 1639 melanoma patients and 1342 controls, revealed that *TYR* variants were associated with risk factors in the European population (OR = 1.50, 95% CI = 1.11–2.04, *p* = 0.0089) [51].

*HERC2* variants (rs916977, rs7170852, and rs12913832) are associated with eye color and UV sensitivity [52,53]. We found that the rs7170852 SNP functions as both a risk factor and a protective factor, depending on the genetic model. In the codominant and recessive models, it acts as a risk factor, whereas in the dominant and overdominant models, it functions as a protective factor. According to Gelmi et al., the rs12913832 variant in the *HERC2* gene was associated with a worse prognosis (*p* = 0.017) in an analysis of 392 patients with melanoma and blue eyes [53]. The *TYRP1* variant (rs1408799) has been associated with blue eyes and melanin production [54,55]. In our study, in the analysis of the log-additive model, the rs1408799 variant was identified as a risk factor (OR = 2.29, *p* = 0.027).

Although *MC1R* variants did not show a significant association in our models, their established influence on melanocyte function suggests that they may contribute to melanoma risk through complex gene–gene and gene–environment interactions [56,57,58,59]. These findings reinforce the biological plausibility of our PRS model and highlight the need for further functional studies to elucidate the mechanisms underlying these associations. This study represents one of the first attempts to assess melanoma risk via a polygenic risk score in a Colombian population.

Despite providing novel insights, our study has several limitations. First, the sample size (250 participants) may limit the statistical power to detect modest associations and fully capture the genetic heterogeneity within the Colombian population. Second, our analysis was restricted to 37 previously reported SNPs, potentially overlooking additional risk variants that contribute to melanoma susceptibility. Third, we acknowledge the issue of multiple testing—multiple SNPs were analyzed under various genetic models—and that nominal *p*-values (*p* < 0.05) may not remain significant after appropriate corrections such as Bonferroni or FDR adjustments. Future studies with larger cohorts should address this to confirm the observed associations. Additionally, our haplotype analysis, which was adjusted only for sex and family history of cancer, lacked detailed reporting on the number of haplotypes tested and the corresponding *p*-values, which limits the interpretability of these findings. Lastly, although our quartile-based stratification of PRS aimed to uncover risk patterns, the distribution of cases across quartiles (with both the lowest and highest quartiles showing an excess of cases) indicates a complex, non-linear relationship that warrants further investigation. Together, these limitations underscore the need for more comprehensive analyses incorporating larger sample sizes, additional covariates, and rigorous statistical corrections to further elucidate the multifactorial basis of melanoma risk in our population.

## 4. Materials and Methods

### 4.1. Ethics Review Board Statement

This study adhered to the ethical guidelines of the Declaration of Helsinki and was approved by the Research Ethics Committee of the Hospital Universitario Centro Dermatologico Federico Lleras Acosta E.S.E. in Bogotá, Colombia (Grant code: 4000.16.6AE).

### 4.2. Study Design and Population

This analytical, observational, case–control study was conducted between 2018 and 2019. A total of 250 participants (85 cases and 165 controls) were recruited after providing informed consent from the Andes region, especially in Bogota. Melanoma cases were selected from patients diagnosed with cutaneous melanoma confirmed by pathology at the tumor clinic of the Hospital Universitario Centro Dermatologico Federico Lleras Acosta E.S.E. For the control group, subjects were also required to be over 18 years of age and to have neither a personal nor a family history of melanoma. Additionally, a thorough physical examination was performed by a dermatologist to rule out any lesions suggestive of melanoma, and the controls were selected from community members or non-family companions of patients consulting for other pathologies. The participants were matched by age, sex, and Fitzpatrick skin phototype. Sociodemographic, morphological, and clinical data were collected during the recruitment period.

### 4.3. DNA Preparation and Single-Nucleotide Polymorphism (SNP) Analysis

Blood samples were collected, and genomic DNA was extracted via a QIAamp^®^ DNA Mini Kit (cat. # 56304 Qiagen, Hilden, Germany) following the manufacturer’s protocol. The DNA concentration was quantified via NanoDrop^®^ equipment (Thermo Fisher Scientific, Waltham, MA, USA). The primers used for conventional, and qPCR were designed to target *MIFT*, *TYP*, *TYRP1*, *OCA2*, *HERC2*, and *MC1R* variants. The primer sequences are detailed in Supplementary Table S1. The PCR conditions followed previous protocols, and the products were validated by gel electrophoresis [12,38].

Fifty randomly selected samples were subjected to Sanger sequencing for initial variant validation. Sequencing was performed via a BigDye Terminator V1.1 Cycle Sequencing Kit (Life Technologies en Carlsbad, CA, USA) and an ABI PRISM 3130xl Genetic Analyzer (Applied Biosystems^®^, Foster City, CA, USA). The results were reviewed with Chromas (free version) and NovoSNP version 3.0 (free version from the internet, https://novosnp.bioinf.be/downloads.html, access on 15 November 2024) software. All the samples were subsequently genotyped via qPCR high-resolution melting (HRM) analysis on a CFX96 Touch^TM^ Real-Time PCR system (Bio-Rad, China). Melt calibration was performed according to the manufacturer’s protocol with a melt calibration kit (cat. #184–5020 Bio-Rad).

The qPCR conditions were as follows: 2 min at 0.5 °C for initial denaturation, 45 cycles of denaturation for 10 s at 95 °C, annealing for 30 s (between 53 °C and 65 °C depending on the exon), and 30 s extension at 72 °C. HRM analysis was subsequently performed as follows: 30 s at 95 °C, 1 min at 60 °C for heteroduplex formation, and 10 s/step at 65–95 °C with 0.2 °C increments for the high-resolution melting cycle. The results were analyzed via Precision Melt Analysis™ v1.1 software, with the sequenced samples used as positive and negative controls. Normalized melting curves were used to classify the genotypes. *CDKN2A*, *CDK4*, and *SCL45A2* variants were genotyped in previous studies and were included in the analysis [12,38,39].

### 4.4. GWAS Databases and Variant Selection

A panel of 37 SNPs located in *CDKN2A*, *CDK4*, *SCL45A2*, *MIFT*, *TYP*, *TYRP1*, *OCA2*, *HERC2*, and *MC1R* genes was selected on the basis of prior studies and publicly available GWAS datasets. Most of these genes have been reported to be associated with melanoma risk in diverse populations. To ensure the relevance of the selection variants, allele frequencies, beta coefficients, and *p* values were extracted from several large GWASs by the international GenoMEL (melanoma genetics consortium, https://genomel.org/, access on 20 December 2024). We downloaded reported GWAS variants from the NHGRI-EBI Catalog of Human Genome-Wide Association Studies (file data: 15 December 2024, https://www.ebi.ac.uk/gwas/home) and the PLCO atlas from the National Cancer Institute (https://exploregwas.cancer.gov/, access on 15 December 2024) (Supplementary Table S2). Variants were prioritized if they had consistent evidence of association in at least two independent studies and demonstrated statistical significance in a population similar to the Colombian population. Details of the selected variants, including their genomic positions, minor allele frequencies, and effect sizes, are provided in Supplementary Table S2.

### 4.5. Polygenic Risk Score Calculation

The PRS was computed to quantify each patient’s cumulative genetic susceptibility to melanoma. It was calculated as a weighted sum, where the number of risk alleles carried by each individual was multiplied by the corresponding beta coefficient. To ensure consistency, we initially retrieved melanoma-associated SNPs from multiple GWAS sources. However, after reviewing the availability of effect sizes, we constructed the final PRS model using beta coefficients exclusively from a single GWAS conducted by the GenoMEL Consortium, reducing potential bias in our analysis (Supplementary Table S2). This approach integrates the effects of multiple genetic variants into a single measure representing an individual’s overall polygenic risk. The participants were stratified into quartiles based on their PRS, enabling comparisons of risk levels between cases and controls.

The PRS was calculated for each patient and controlled via the following formula:

PRS = β_1_x_1_ + β_2_x_2_ + β_3_x_3_ + …… + β_37_x_37_

where β is the beta value obtained from the GWAS and where x represents the number of risk alleles per SNP (0, 1, or 2). To assess the predictive performance of the PRS, receiver operating characteristic (ROC) curves were generated, and the area under the curve (AUC) was calculated to evaluate its discriminatory capacity. The PRS analysis was performed compared cases and controls, according to melanoma subtypes, and according to acral and no acral melanoma. To estimate the phenotypic variance explained by the selected SNPs, we fitted a logistic regression model with melanoma status as the outcome and PRS as the predictor. The explanatory power of this model was quantified using the Nagelkerke pseudo R^2^, computed with the pR2 function from the pscl v-1.5.9package in R v-4-4-1.

### 4.6. Statistical Analysis

Statistical analyses were performed in R studio and SNPStats tool (http://bioinfo.iconcologia.net/SNPstats, access on 15 November 2024) from the Catalan Institute of Oncology in Barcelona, Spain. For each SNP, allele, and genotype, frequencies were calculated, and the Hardy–Weinberg equilibrium (HWE) was assessed in the control group via a chi-square test (χ^2^). Deviations from HWE were considered significant at *p* < 0.05. The association between each SNP and melanoma risk was evaluated under various genetic models, including dominant, recessive, codominant, additive, and overdominant models. Logistic regression was used to estimate odds ratios (ORs) and 95% confidence intervals (CIs) for each model. The design used for these analyzes was unmatched. Haplotype analysis was conducted for SNPs located within the same gene or genomic region, providing additional information on the combined effects of alleles in linkage disequilibrium. Haplotypes were inferred using the expectation–maximization (EM) algorithm implemented in the Haplo.stats package (version 1.7.7) in R-Studio. Additionally, SNPStats was used to verify and cross-check haplotype inference results. Both tools utilize the EM algorithm for haplotype construction and subsequent association testing with melanoma risk using logistic regression. Rare haplotypes with frequencies below 1% were excluded from the analysis to ensure statistical power.

The PRS was incorporated into a multivariate logistic regression model along with clinical and pathological characteristics to evaluate its combined effect on melanoma susceptibility. Statistical significance was set at *p* < 0.05, and all analyses were performed via RStudio (version 4.2.3) software.

## 5. Conclusions

In conclusion, our study demonstrates the feasibility and potential clinical utility of using a polygenic risk score approach to assess melanoma risk in a Colombian population. By integrating genetic risk scores with traditional clinical factors, our model shows promise for improving early detection and prevention strategies. This comprehensive approach not only captures the multifactorial nature of melanoma risk more effectively than single-gene analyses do but also offers a valuable tool for tailoring screening and intervention programs to the unique genetic landscape of the Colombian population.

## Figures and Tables

**Table 1:** Polygenic risk score (PRS) quartile distribution by melanoma risk. *p* value from Kruskal–Wallis test.

PRS Quartile	Cases (85)		Controls (165)		Proportion	(95% CI)	*p* Value	
	Frequency	%	Frequency	%				
Q1	29	34.1	34	20.6	0.46	0.336–0.590	0.6140	
Q2	15	17.6	47	28.5	0.242	0.146–0.370	0.0001	***
Q3	14	16.5	48	29.1	0.226	0.133–0.353	0.0000	****
Q4	27	31.8	36	21.8	0.429	0.307–0.559	0.3130	

Significance codes: 0 ‘****’, 0.001 ‘***’.

**Table 2:** Clinical characteristics of the study population based on the quartile stratification.

		PRS Quartile 1				PRS Quartile 2				PRS Quartile 3				PRS Quartile 4			
		Cases (29)		Controls (34)		Cases (15)		Controls (47)		Cases (14)		Controls (48)		Cases (27)		Controls (36)	
		N°	%	N°	%	N°	%	N°	%	N°	%	N°	%	N°	%	N°	%
Sex	Female	17	58.62	18	52.94	7	46.67	26	55.32	8	57.14	29	60.42	14	51.85	16	44.44
	Men	12	41.38	16	47.06	8	53.33	21	44.68	6	42.86	19	39.58	13	48.15	20	55.56
Phototype	2	4	13.79	5	14.71	2	13.33	8	17.02	5	35.71	12	25.00	5	18.52	6	16.67
	3	21	72.41	26	76.47	11	73.33	31	65.96	7	50.00	35	72.92	20	74.07	25	69.44
	4	4	13.79	3	8.82	2	13.33	8	17.02	2	14.29	1	2.08	2	7.41	5	13.89
Pathologic diagnostic	Acral lentiginous melanoma	9	31.03	-	-	2	13.33	-	-	4	28.57	-	-	5	18.52	-	-
	Lentigo maligna melanoma	11	37.93	-	-	5	33.33	-	-	4	28.57	-	-	11	40.74	-	-
	Nodular melanoma	3	10.34	-	-	2	13.33	-	-	3	21.43	-	-	5	18.52	-	-
	Superficial spreading melanoma	6	20.69	-	-	6	40.00	-	-	3	21.43	-	-	5	18.52	-	-
Clark grade	<1 mm	1	3.45	-	-	1	6.67	-	-	0	0.00	-	-	0	0.00	-	-
	2 mm	1	3.45	-	-	2	13.33	-	-	1	7.14	-	-	2	7.41	-	-
	3 mm	1	3.45	-	-	0	0.00	-	-	1	7.14	-	-	0	0.00	-	-
	>4 mm	13	44.83	-	-	5	33.33	-	-	7	50.00	-	-	15	55.56	-	-
	Negative	13	44.83	-	-	7	46.67	-	-	5	35.71	-	-	8	29.63	-	-
Breslow scale	≤1.0 mm	4	13.79	-	-	1	6.67	-	-	2	14.29	-	-	4	14.81	-	-
	>1.0–2.0 mm	1	3.45	-	-	2	13.33	-	-	1	7.14	-	-	5	18.52	-	-
	>2.0–4.0 mm	5	17.24	-	-	2	13.33	-	-	1	7.14	-	-	4	14.81	-	-
	>4.0 mm	7	24.14	-	-	3	20.00	-	-	5	35.71	-	-	6	22.22	-	-
	Non reported	12	41.38	-	-	7	46.67	-	-	5	35.71	-	-	8	29.63	-	-
Location	Head and neck	12	41.38	-	-	8	53.33	-	-	3	21.43	-	-	14	51.85	-	-
	Trunk	5	17.24	-	-	1	6.67	-	-	3	21.43	-	-	3	11.11	-	-
	Upper extremities	2	6.90	-	-	0	0.00	-	-	1	7.14	-	-	2	7.41	-	-
	Lower extremities	1	3.45	-	-	4	26.67	-	-	3	21.43	-	-	2	7.41	-	-
	Hands and Feets	9	31.03	-	-	2	13.33	-	-	4	28.57	-	-	6	22.22	-	-
Eye colors	Black or dark brown	14	48.28	24	70.59	10	66.67	36	76.60	6	42.86	33	68.75	14	51.85	28	77.78
	Blue	1	3.45	2	5.88	1	6.67	2	4.26	0	0.00	3	6.25	0	0.00	0	0.00
	Light brown	10	34.48	7	20.59	3	20.00	7	14.89	6	42.86	9	18.75	11	40.74	4	11.11
	Green	4	13.79	1	2.94	1	6.67	1	2.13	2	14.29	3	6.25	2	7.41	4	11.11
Hair colors	Black or dark brown	21	72.41	28	82.35	12	80.00	39	82.98	7	50.00	34	70.83	15	55.56	34	94.44
	Light brown	8	27.59	5	14.71	3	20.00	8	17.02	7	50.00	13	27.08	11	40.74	2	5.56
	Red	0	0.00	1	2.94	0	0.00	0	0.00	0	0.00	1	2.08	1	3.70	0	0.00
Familiar cancer history	Yes	17	58.62	16	47.06	7	46.67	19	40.43	7	50.00	27	56.25	17	62.96	16	44.44
	No	12	41.38	18	52.94	8	53.33	28	59.57	7	50.00	21	43.75	10	37.04	20	55.56

**Table 3:** Estimated effect size by patient features and quartile stratification.

	PRS Quartile	Cases (85)	Controls (165)	X^2^	OR	(95% CI)	*p* Value	
Sex (Female)	Q1	17	18	0.20	1.25	0.46–3.42	0.651	
	Q2	7	26	0.34	0.71	0.22–2.26	0.558	
	Q3	8	29	0.05	0.87	0.26–2.91	0.826	
	Q4	14	16	0.33	1.35	0.49–3.66	0.560	
Phototype (3)	Q1	21	26	0.13	0.80	0.25–2.51	0.710	
	Q2	11	31	0.28	1.41	0.38–5.17	0.590	
	Q3	7	35	2.60	0.37	0.10–1.26	0.106	
	Q4	20	25	0.16	1.25	0.41–3.83	0.680	
Eye Color								
Black or dark brown	Q1	14	24	3.25	0.38	0.13–1.09	0.070	
	Q2	10	36	0.58	0.61	0.17–2.17	0.440	
	Q3	6	33	3.11	0.34	0.100–1.15	0.070	
	Q4	14	28	4.66	0.30	0.10–0.91	0.031	*
Light brown	Q1	10	7	1.53	2.03	0.65–6.28	0.210	
	Q2	3	7	0.21	1.42	0.31–6.39	0.630	
	Q3	6	9	3.43	3.25	0.90–11.72	0.063	
	Q4	11	4	7.46	5.50	1.51–20.02	0.006	**
Hair colors								
Black or dark brown	Q1	21	28	0.89	0.56	0.16–1.86	0.340	
	Q2	12	39	0.06	0.82	0.18–3.59	0.790	
	Q3	7	34	2.10	0.41	0.12–1.39	0.140	
	Q4	15	34	13.50	0.07	0.01–0.36	0.000	****
Light brown	Q1	8	5	1.58	2.20	0.63–7.71	0.208	
	Q2	3	8	0.06	1.21	0.27–5.33	0.790	
	Q3	7	13	2.60	2.69	0.79–9.17	0.106	
	Q4	11	2	11.66	11.68	2.31–59.03	0.001	***

Significance codes: 0 ‘****’, 0.001 ‘***’, 0.01 ‘**’, 0.05 ‘*’.

**Table 4:** Genotype and allele distributions of variants in different genes across cases and controls.

Model	Genotype Allele	Cases *n* = 85 (%)	Control *n* = 166 (%)	OR	(95% CI)	*p* Value
TYR (rs1042602—S192Y)						
Codominant	C/C	46 (54.1%)	74 (44.9%)	1	Reference	
	C/A	35 (41.2%)	66 (40%)	1.08	(0.58–2.01)	0.096
	A/A	4 (4.7%)	25 (15.2%)	3.44	(1.01–11.70)	
Dominant	C/C	46 (54.1%)	74 (44.9%)	1.32	(0.73–2.38)	0.36
	C/A-A/A	39 (45.9%)	91 (55.1%)			
Recessive	C/C-C/A	81 (95.3%)	140 (84.8%)	3.32	(1.01–10.94)	0.032
	A/A	4 (4.7%)	25 (15.2%)			
Overdominant	C/C-A/A	50 (58.8%)	99 (60%)	0.90	(0.49–1.64)	0.73
	C/A	35 (41.2%)	66 (40%)			
Log-additive				1.46	(0.92–2.31)	0.1
TYRP1 (rs1408799)						
Codominant	T/T	70 (82.3%)	123 (74.5%)	1	Reference	
	T/C	15 (17.6%)	41 (24.9%)	2.04	(0.92–4.51)	0.049
	C/C	0 (0%)	1 (0.6%)	NA	(NA)	
Dominant	T/T	70 (82.3%)	123 (74.5%)	2.19	(0.99–4.83)	0.044
	T/C-C/C	15 (17.6%)	42 (25.4%)			
Recessive	T/T-T/C	85 (100%)	164 (99.4%)	NA	(0.00–NA)	-
	C/C	0 (0%)	1 (0.6%)			
Overdominant	T/T-C/C	70 (82.3%)	124 (75.2%)	1.97	(0.90–4.33)	0.08
	T/C	15 (17.6%)	41 (24.9%)			
Log-additive				2.29	(1.07–4.90)	0.027
HERC2 (rs7170852—Intron56)						
Codominant	T/T	34 (40%)	89 (53.9%)	1	Reference	
	T/A	50 (58.8%)	42 (25.4%)	0.28	(0.14–0.55)	<0.0001
	A/A	1 (1.2%)	34 (20.6%)	12.22	(1.48–100.91)	
Dominant	T/T	34 (40%)	89 (53.9%)	0.52	(0.28–0.98)	0.04
	T/A-A/A	51 (60%)	76 (46.1%)			
Recessive	T/T-T/A	84 (98.8%)	131 (79.4%)	21.92	(2.73–176.14)	<0.0001
	A/A	1 (1.2%)	34 (20.6%)			
Overdominant	T/T-A/A	35 (41.2%)	123 (74.5%)	0.20	(0.10–0.39)	<0.0001
	T/A	50 (58.8%)	42 (25.4%)			
Log-additive				1.11	(0.72–1.70)	0.65

NA: Analysis is not performed due to low frequency.

## Data Availability

The files related to the informed consent, clinical data, and molecular biology results and the data used to support the findings of this study are restricted by the ethics committee of the Hospital Universitario—Centro Dermatologico Federico Lleras Acosta E.S.E., DC, Bogota, Colombia. However, the data are available from file code 1DSI02-6AE for researchers who meet the criteria for access to confidential data. These files can be requested from the ethics committee using the following email: comitedeeticaeninvestigacion@dermatologia.gov.co.

## Supplementary Materials

The following supporting information can be downloaded at https://www.mdpi.com/article/10.3390/ijms26104674/s1.

## Abbreviations

The following abbreviations are used in this manuscript:

PRS	Polygenic risk score
GWAS	Genome-wide association study
Q	Quartile
SNP	Single-nucleotide polymorphisms
AUC	Area under the curve
OR	Odds ratio
ROC	Receiver operating characteristic
CI	Confidence intervals
HWE	Hardy–Weinberg equilibrium
HRM	High-resolution melting
